# P2X7 receptor activation mediates superoxide dismutase 1 (SOD1) release from murine NSC-34 motor neurons

**DOI:** 10.1007/s11302-022-09863-5

**Published:** 2022-04-28

**Authors:** Rachael Bartlett, Diane Ly, Neil R. Cashman, Ronald Sluyter, Justin J. Yerbury

**Affiliations:** 1 Illawarra Health and Medical Research Institute, Wollongong, NSW 2522 Australia; 2 Molecular Horizons and School of Chemistry and Molecular Bioscience, University of Wollongong, Wollongong, NSW 2522 Australia; 3 Djavad Mowafaghian Centre for Brain Health, University of British Columbia, Vancouver, BC V6T 1Z3 Canada

**Keywords:** Motor neuron disease, Purinergic receptor, Glia, Neuroinflammation, Protein aggregation, Protein misfolding

## Abstract

Mutant superoxide dismutase 1 (SOD1) can be constitutively released from motor neurons and transmitted to naïve motor neurons to promote the progression of amyotrophic lateral sclerosis (ALS). However, the biological impacts of this process and the precise mechanisms of SOD1 release remain to be fully resolved. Using biochemical and fluorescent techniques, this study aimed to determine if P2X7 receptor activation could induce mutant SOD1 release from motor neurons and whether this released SOD1 could be transmitted to motor neurons or microglia to mediate effects associated with neurodegeneration in ALS. Aggregated SOD1^G93A^, released from murine NSC-34 motor neurons transiently transfected with SOD1^G93A^, could be transmitted to naïve NSC-34 cells and murine EOC13 microglia to induce endoplasmic reticulum (ER) stress and tumour necrosis factor-alpha (TNFα) release, respectively. Immunoblotting revealed NSC-34 cells expressed P2X7. Extracellular ATP induced cation dye uptake into these cells, which was blocked by the P2X7 antagonist AZ10606120, demonstrating these cells express functional P2X7. Moreover, ATP induced the rapid release of aggregated SOD1^G93A^ from NSC-34 cells transiently transfected with SOD1^G93A^, a process blocked by AZ10606120 and revealing a role for P2X7 in this process. ATP-induced SOD1^G93A^ release coincided with membrane blebbing. Finally, aggregated SOD1^G93A^ released via P2X7 activation could also be transmitted to NSC-34 and EOC13 cells to induce ER stress and TNFα release, respectively. Collectively, these results identify a novel role for P2X7 in the prion-like propagation of SOD1 in ALS and provide a possible explanation for the therapeutic benefits of P2X7 antagonism previously observed in ALS SOD1^G93A^ mice.

**Supplementary Information:**

The online version contains supplementary material available at 10.1007/s11302-022-09863-5.

## Introduction

Amyotrophic lateral sclerosis (ALS) is a fatal neurodegenerative disease characterised by the loss of upper and lower motor neurones and ensuing muscle dysfunction including respiratory paralysis [1]. Neuroanatomically, ALS spreads in a contiguous fashion commencing in a focal region before spreading to neighbouring motor neurons and then other regions of the nervous system [2]. This spreading is thought to involve the prion-like propagation of protein misfolding and aggregation between neighbouring cells [3].

Accumulating evidence suggests a role for mutant superoxide dismutase 1 (SOD1) in the prion-like propagation in ALS [4], a protein long-known to be associated with ALS development [5, 6] and with at least 155 mutations associated with the familial form of this disease [7]. In vitro studies reveal that mutant SOD1 aggregates released from neural cells can be internalised by other neural cells via macropinocytosis to seed the aggregation of intracellular, soluble mutant SOD1 [8], and wild-type SOD1 (SOD1^WT^) [9]. Once transmission has commenced, SOD1 aggregates are readily transferred from cell to cell in a contact-independent manner [8]. Subsequent studies have confirmed that the internalisation of SOD1 aggregates is mediated via macropinocytosis [10, 11]. Notably, in vivo studies demonstrate the prion-like propagation of SOD1 aggregates initiated by injection of either aggregated recombinant mutant SOD1 or SOD1^WT^ [12, 13] or homogenates from ALS SOD1^G85R^ or SOD1^G93A^ mice [14] or ALS patients [12] into ALS SOD1^G85R^ mice. In vitro studies also show that mutant SOD1 and SOD1^WT^ aggregates can be released from cells via either cell death or exosomes [10], the latter consistent with an earlier report demonstrating mutant SOD1 release via exosomes from murine NSC-34 motor neuron cells [15]. However, other mechanisms of SOD1 release may exist [10] and the precise mechanisms of SOD1 release in vivo and the triggers underpinning these processes are unknown.

In addition to the prion-like propagation of protein misfolding and aggregation, uptake of aggregated mutant SOD1 can initiate cellular events similar to those associated with neurodegeneration in ALS. Uptake of aggregated recombinant mutant SOD1 into human SH-SY5Y neuroblastoma cells induces endoplasmic reticulum (ER) stress [16]. Likewise, uptake of aggregated recombinant mutant SOD1 into murine EOC13 microglia can induce TNFα release [17]. Whether aggregated mutant SOD1 released from cells can induce similar cellular events to aggregated recombinant SOD1 remains unknown.

The P2X7 receptor is a trimeric ligand-gated ion channel/pore activated by extracellular adenosine 5′-triphosphate (ATP) [18, 19] and is present on various immune and neural cell types [20, 21]. The receptor has emerging roles in the development of ALS [22, 23], and its pharmacological blockade can limit disease progression in ALS SOD1^G93A^ mice [24–27]. The role of P2X7 in ALS, however, is yet to be fully established. Notably, P2X7 activation can induce the release of proteins from various cell types [28–30] and in neural cells can induce exosome release [30] and cell death [31]. Collectively, this suggests that P2X7 activation could potentially be involved in the release of aggregated mutant SOD1 from motor neurons.

Using murine NSC-34 motor neurons transfected with human SOD1^G93A^, this study aimed to determine whether aggregated SOD1^G93A^ released from these cells could be transmitted to naïve NSC-34 cells and murine EOC13 microglia to induce ER stress and TNFα release, respectively. Then, using SOD1^G93A^-transfected NSC-34 cells, this study aimed to determine if P2X7 activation could induce the release of aggregated SOD1^G93A^ from these cells and whether this released SOD1^G93A^ could also induce ER stress in NSC-34 cells and TNFα release from EOC13 cells.

## Materials and methods

### Cell culture

Murine neuroblastoma × spinal cord hybrid NSC-34 cells have been described elsewhere [32]. Murine lymphoblast LADMAC, murine microglial EOC13, and human embryonic kidney (HEK)-293 cells were from the American Type Culture Collection (Manassas, VA, USA). NSC-34 and LADMAC cells were maintained in DMEM/F12 medium (Life Technologies, Carlsbad, CA, USA) supplemented with 10% heat-inactivated FBS (Bovogen Biologicals, Keilor East, Australia) and 2 mM GlutaMAX (Life Technologies) (complete medium). EOC13 cells were maintained in complete medium supplemented with 20% LADMAC conditioned medium. HEK-293 cells were maintained in complete medium supplemented with 100 U/mL penicillin and 100 μg/mL streptomycin (Life Technologies). Cells were maintained at 37 °C/5% CO_2_ and harvested with 0.05% trypsin/0.5 mM EDTA (Life Technologies).

### Plasmid purification

pEGFP-N1 expression vectors containing SOD1^WT^ and SOD1^G93A^ cDNAs were kindly provided by Bradley Turner (University of Melbourne, Melbourne, Australia) [33]. pFUW expression vectors containing SOD1^G85R^ and SOD1^G127X^ cDNAs were kindly provided by Edward Pokrishevsky (University of British Columbia, Vancouver, Canada) [9]. Chemically competent DH5α *Escherichia coli*, kindly provided by Jason McArthur (University of Wollongong, Wollongong, Australia), were transformed with SOD1-containing plasmids using heat shock (42 °C) and colonies resistant to kanamycin (Ameresco, Framingham, MA, USA) selected. Plasmid DNA was purified using the CompactPrep Plasmid Maxi Kit (Qiagen, Hilden, Germany) as per the manufacturer’s instructions.

### Transfection of NSC-34 cells

NSC-34 cells in complete medium in 6-well plates (7 × 10^5^ cells/well) or 12-well plates (3 × 10^5^ cells/well) were incubated overnight at 37 °C/5% CO_2_. NSC-34 cells were washed once with DMEM/F12 medium and incubated for 5 h in DMEM/F12 medium containing 0.5 μg plasmid DNA and Lipofectamine 2000 (Life Technologies), or 1.5 μg plasmid DNA and Lipofectamine LTX (Life Technologies), respectively. Cells were then incubated in complete medium for a further 67 h (72 h total). Alternatively, NSC-34 cells were incubated in complete medium containing 2.5 μg plasmid DNA, P3000 reagent, and Lipofectamine 3000 (Life Technologies) for 72 h. Transfection efficiency of EGFP-containing vectors was confirmed by fluorescence microscopy using an Eclipse TE2000 inverted microscope (Nikon, Tokyo, Japan) and was similar between vectors, typically ranging from 17 to 27%. Transiently transfected NSC-34 cells were used at 72 h in subsequent assays.

### Constitutive SOD1 release

Conditioned media were collected from transiently transfect NSC-34 cells at 72 h and centrifuged (20,000 × *g* for 30 min) to obtain pelletable fractions. Pelletable fractions were then resuspended as described for use in subsequent assays.

### Detection of cell-associated EGFP by flow cytometry

Pelletable fractions from treated NSC-34 cells (as indicated) were resuspended in 500 µL complete medium and incubated with naïve NSC-34 or EOC13 cells in 24-well plates (5 × 10^4^ cells/well) for 24 h at 37 °C/5% CO_2_. Cells were visualised by differential interference contrast imaging using an Eclipse TE2000 inverted microscope. Cells were then harvested, washed once with PBS and data acquired using a BD (San Diego, CA, USA) LSR II flow cytometer. The percentage of EGFP^+^ cells was assessed using FlowJo 8.7.1 (Tree Star, Ashland, OR, USA).

### ER stress assay

Pelletable fractions from treated NSC-34 cells (as indicated) were resuspended in 500 µL complete medium and incubated with NSC-34 transiently transfected with X-box-binding protein 1 (XBP1)-tdTomato in 24-well plates (5 × 10^4^ cells/well) for 24 h at 37 °C/5% CO_2_. Cells were visualised by differential interference contrast and fluorescence imaging using an Eclipse TE2000 inverted microscope with images captured using Image-Pro AMS 6.1 (Media Cybernetics, Rockville, MD, USA) or using an IncuCyte ZOOM Live-cell Analysis System (Essen BioScience, Ann Arbor, MI) with images captured using IncuCyte ZOOM software (Essen BioScience). Cells were then harvested, washed once with PBS and data acquired using a LSR II flow cytometer. The mean fluorescence intensity (MFI) of relative XBP1-tdTomato fluorescence was determined using FlowJo 8.7.1. Alternatively, cells in plates were visualised using a Leica (Wetzlar, Germany) DM IBRE inverted microscope and Leica TCS SP confocal imaging system.

### TNFα release assay

Pelletable fractions from treated NSC-34 cells (as indicated) were resuspended in 500 µL complete medium and incubated with naïve EOC13 cells in 24-well plates (5 × 10^4^ cells/well) for 24 h at 37 °C/5% CO_2_. Media from wells were then centrifuged (20,000 × *g* for 5 min at 4 °C) and cell-free supernatants stored at − 80 °C until required. The presence of TNFα measured using a Murine TNF-α ELISA Ready-SET-Go! Kit (eBioscience, San Diego, CA, USA) as per the manufacturer’s instructions.

### Detection of P2X7 by immunoblotting

Proteins in lysates of NSC-34, EOC13 and P2X7-transfected HEK-293 cells were separated under reducing conditions using Any kD Mini-PROTEAN TGX Stain-Free gels (Bio-Rad, Hercules, CA, USA) and transferred to nitrocellulose membranes (Bio-Rad), and P2X7 detected by immunoblotting using a rabbit anti-murine P2X7 polyclonal antibody (Cat. No. APR-008, Alomone Labs, Jerusalem, Israel) as described [34].

### ATP-induced uptake assay

Non-transfected or transiently transfected NSC-34 cells in 6-well plates (7 × 10^5^ cells/mL) were washed twice with DMEM/F12 medium and pre-incubated in the absence or presence of 10 μM AZ10606120 (Tocris Bioscience, Bristol, UK) for 1 h at 37 °C. Cells were then incubated with 25 μM ethidium bromide (Ameresco) in the absence (basal) or presence of 5 mM ATP (Sigma-Aldrich, St Louis, MO, USA) in DMEM/F12 medium for 20 min at 37 °C. Reactions were stopped by the addition of an equal volume of ice-cold DMEM/F12 medium containing 20 mM MgCl_2_. Cells were then harvested and washed once with DMEM/F12 medium. Data was acquired using a BD LSR II flow cytometer. The MFI of relative ethidium^+^ uptake was assessed using FlowJo 8.7.1.

### ATP-induced SOD1 release assay

Transiently transfected NSC-34 cells in 12-well plates (3 × 10^5^ cells/mL) were washed twice with DMEM/F12 medium and pre-incubated in the absence or presence of 10 µM AZ10606120 in DMEM/F12 medium for 1 h at 37 °C/5% CO_2_. Cells were then incubated in the absence (basal) or presence of ATP (as indicated) for 20 min at 37 °C. Following incubation in the absence or presence of ATP, medium was collected and centrifuged (20,000 × *g* for 30 min) to obtain pelletable fractions.

### Detection of SOD1 by immunoblotting

Pelletable fractions from treated NSC-34 cells (as indicated), resuspended in equal volumes of sample buffer containing 5% 2-mercaptoethanol (Ameresco) were separated using Any kD Mini-PROTEAN TGX Stain-Free gels and transferred to nitrocellulose membranes. In some experiments, gels were imaged, prior to transfer, using a Bio-Rad Criterion Stain Free Gel Imaging System. Membranes were blocked with either 1% heat denatured casein (Sigma-Aldrich) in PBS (for EGFP) or Tris-buffered saline (TBS; 250 mM NaCl and 50 mM Tris, pH 7.5) containing 0.2% Tween-20 and 5% milk powder (for SOD1) for 1 h. Membranes were incubated with either rabbit anti-EGFP polyclonal antibody (Cat. No. ab290 or ab6556, Abcam, Cambridge, UK) or rabbit anti-SOD1 (Cat. No. abADI-SOD-100, Enzo Life Sciences, Farmingdale, NY, USA) or sheep anti-SOD1 (Cat. No. ab8866, Abcam) polyclonal antibody (depending on availability) in corresponding blocking solution overnight at 4 °C. Membranes were washed three times with PBS containing 0.1% Triton X-100 (for EGFP) or TBS containing 0.2% Tween-20 (for SOD1). Membranes were then incubated with peroxidise-conjugated anti-rabbit (Cat. No. 1706515, Bio-Rad or Cat. No. NA934, Cytiva, Marlborough, MA, USA) or anti-sheep (Cat. No. AB324P, Merck Millipore, Burlington, MA, USA) IgG antibody in corresponding blocking solution for 1 h. Membranes were washed and visualised using SuperSignal West Pico Chemiluminescence Substrate (ThermoFisher Scientific, Waltham, MA, USA). Relative EGFP and SOD1 were quantified from captured images using ImageJ software 1.48 [35].

### Detection of SOD1 by transmission electron microscopy

Pelletable fractions (20,000 × *g* for 30 min) from treated NSC-34 cells (as indicated) were washed and resuspended in PBS. Fractions (2 µL) were then loaded onto carbon-coated nickel grids (ProSciTech, Kirwan, Australia) and incubated for 1 min. Grids were washed 3–5 times with Milli-Q water. In some experiments, grids were blocked for 40 min with 1% heat denatured casein in PBS, followed by incubation with anti-EGFP polyclonal antibody (Cat. No. ab290, Abcam) or an equivalent amount of rabbit IgG (Cat. No. ab171870, Abcam) for 1 h. Grids were washed three times with 1% heat denatured casein in PBS and incubated with 5 nm gold-conjugated anti-rabbit IgG antibody (Cat. No. ab27235, Abcam) 1 h. Grids were washed as above, followed by five washes with Milli-Q water (Merck Millipore). Non-labelled and labelled grids were stained with Milli-Q water containing 2% uranyl acetate (ProSciTech) for 3 min and dried by blotting. Structures were visualised using a JEOL (Akishima, Japan) JEM2011 transmission electron microscope. Images were captured using Gatan Microscopy Suite Version 2.30.542.0 (Pleasanton, CA, USA).

### Lactate dehydrogenase release assay

Transiently transfected NSC-34 cells in 6-well plates (7 × 10^5^ cells/well) were washed twice with DMEM/F12 medium and pre-incubated in the absence or presence of 10 µM AZ10606120 in DMEM/F12 medium for 1 h at 37 °C/5% CO_2_. Cells were then incubated in the absence (basal) or presence of 5 mM ATP in DMEM/F12 medium for 20 min at 37 °C. Cells were visualised by differential interference contrast imaging using an Eclipse TE2000 inverted microscope with images captured using Image-Pro AMS 6.1. The media from wells were collected and the remaining adherent cells lysed with Lysis Solution (Roche, Mannheim, Germany). Lactate dehydrogenase (LDH) release and percent cytotoxicity were determined using the Cytotoxicity Detection KitPLUS (Roche) as per the manufacturer’s instructions using a Molecular Devices (San Jose, CA, USA) SpectraMax Plus 384 Microplate Reader. The percentage of LDH release was determined as the amount of LDH released into the medium over the total amount of LDH released in each sample.

### Real-time imaging of ATP-induced SOD1 release

Transiently transfected NSC-34 cells in 8-chamber µ-slides (Ibidi, Grafeling, Germany) (5 × 10^5^ cells/0.2 mL/chamber) were washed twice with DMEM/F12 medium and pre-incubated in DMEM/F12 medium at 37 °C for 1 h. Cells with fluorescent aggregates were identified using a DM IBRE inverted microscope and TCS SP confocal imaging system at 37 °C. Cells were then incubated in the absence (basal) or presence of 5 mM ATP for 20 min at 37 °C with fluorescent and bright-field images captured every 30 s with Leica confocal software.

### Detection of cell-associated EGFP by confocal microscopy

Pelletable fractions (20,000 × *g* for 30 min) from treated NSC-34 cells (as indicated) were resuspended in complete medium and incubated with naïve EOC13 cells in 8-chamber µ-slides (5 × 10^5^ cells/0.2 mL/chamber) for 2 or 24 h at 37 °C/5% CO_2_. Cells were visualised by a DM IBRE inverted microscope and TCS SP confocal imaging system. Fluorescent and bright-field 30-step z-stacks were captured using Leica confocal software, and either compressed to form single images, or orthogonally sectioned to confirm that fluorescent particles present were intracellular.

### Data presentation and statistical analyses

Data are presented as the mean ± SD. Differences between multiple groups were compared using a one-way ANOVA with Tukey’s multiple comparison test. Differences between two groups were compared using a two-tailed unpaired Student’s *t*-tests. Statistical analyses were performed using GraphPad Version 8.4.0 (San Diego, CA, USA) with *p* < 0.05 considered statistically significant.

## Results

### SOD1^G93A^ constitutively released from NSC-34 motor neurons induces ER stress in recipient NSC-34 motor neurons

**Fig. 1:**
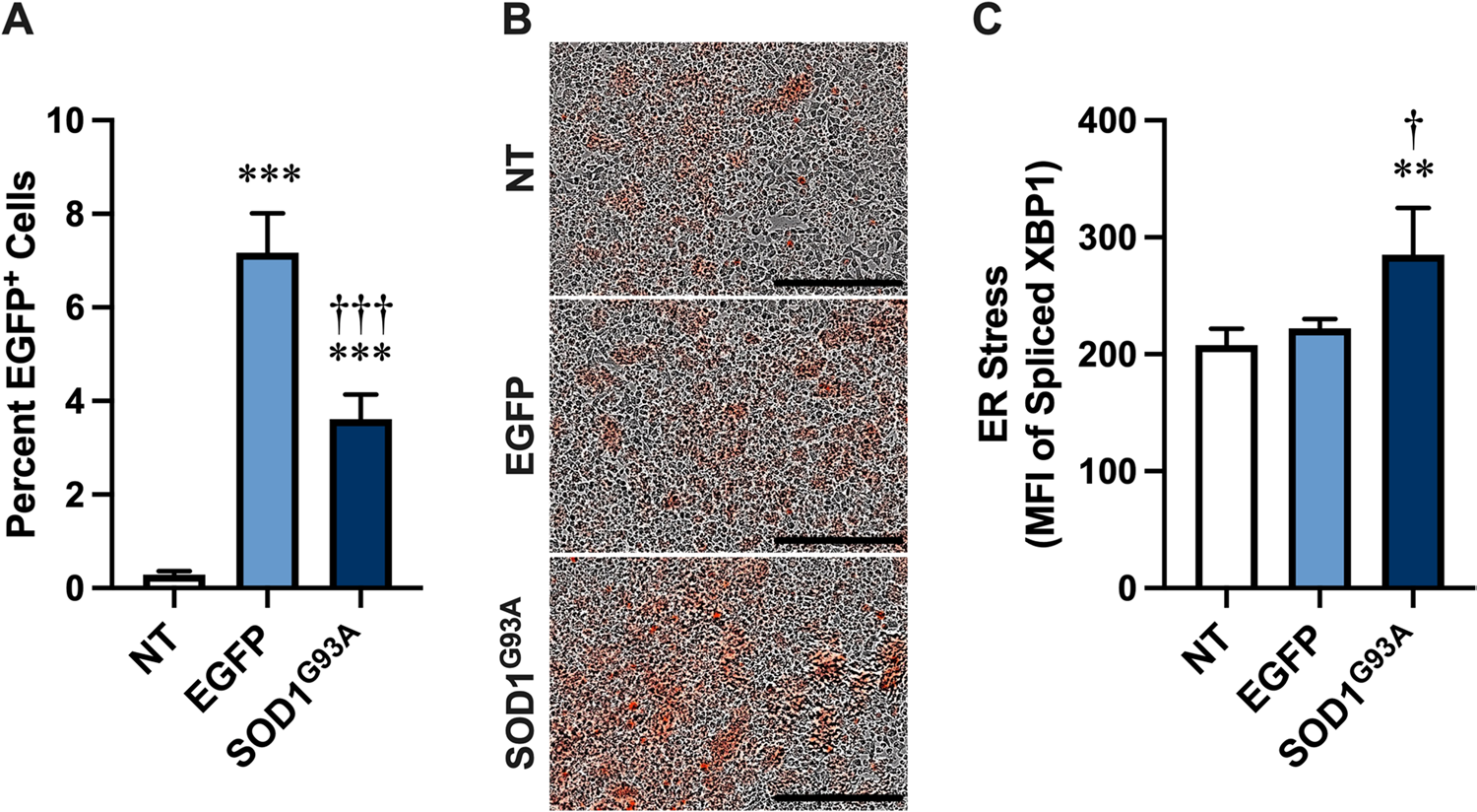
SOD1^G93A^ constitutively released from NSC-34 motor neurons induces ER stress in recipient NSC-34 motor neurons. **A**–**C** XBP1-tdTomato-transfected NSC-34 cells were incubated with pelletable fractions from conditioned media of non-transfected (NT), or EGFP- or SOD1^G93A^-EGFP (SOD1^G93A^)-transfected NSC-34 cells. At 24 h, **A** the percentage of EGFP^+^ cells was assessed by flow cytometry, **B** cellular XBP1-tdTomato fluorescence was visualised by an IncuCyte ZOOM Live-cell Analysis System, and **C** the MFI of XBP1-tdTomato in cells was assessed by flow cytometry. **A**, **C** Data are presented as mean ± SD (*n* = 4); **p < 0.02, ***p < 0.01 compared to NT, ^†^*p* < 0.05, ^†††^*p* < 0.01 compared to EGFP. **B** Data are representative of *n* = 4 experiments; bars represent 300 µm

Aggregated SOD1^G93A^ released from NSC-34 motor neurons can be internalised by naïve NSC-34 cells [10]. Furthermore, uptake of aggregated SOD1^G93A^ into human SH-SY5Y neuroblastoma cells can induce ER stress [16]. To determine if uptake of aggregated SOD1^G93A^ released from NSC-34 cells could similarly induce ER stress in naïve NSC-34 cells, NSC-34 cells were transiently transfected with the ER stress reporter, transcription factor XBP1-tdTomato [36, 37], and incubated for 24 h in the presence of pelletable fractions obtained by centrifugation (20,000 × *g* for 30 min) of conditioned media (72 h) from non-transfected, and EGFP and SOD1^G93A^-EGFP transfected NSC-34 cells and assessed by flow cytometry and fluorescent microscopy. Flow cytometry revealed that a small proportion of cells incubated with pelletable fractions from EGFP and SOD1^G93A^-EGFP transfected cells were positive for EGFP, which was significantly greater than in those cells incubated with pelletable fractions from non-transfected cells (Fig. 1A). Fluorescent microscopy demonstrated the presence of a larger proportion of XBP1-tdTomato fluorescent cells following incubation of XBP1-tdTomato-transfected NSC-34 cells with pelletable fractions from EGFP and SOD1^G93A^-EGFP transfected cells compared to those cells incubated with pelletable fractions from non-transfected cells (Fig. 1B). Notably, flow cytometry revealed a higher amount of fluorescence in cells incubated with pelletable fractions from SOD1^G93A^-EGFP transfected cells compared to those cells incubated with pelletable fractions from either non-transfected or EGFP transfected cells (Fig. 1C). Together these results indicate that SOD1^G93A^ released from NSC-34 cells can associate with and promote ER stress in recipient NSC-34 cells.

### SOD1^G93A^ constitutively released from NSC-34 motor neurons associates with EOC13 microglia to promote TNFα release

**Fig. 2:**
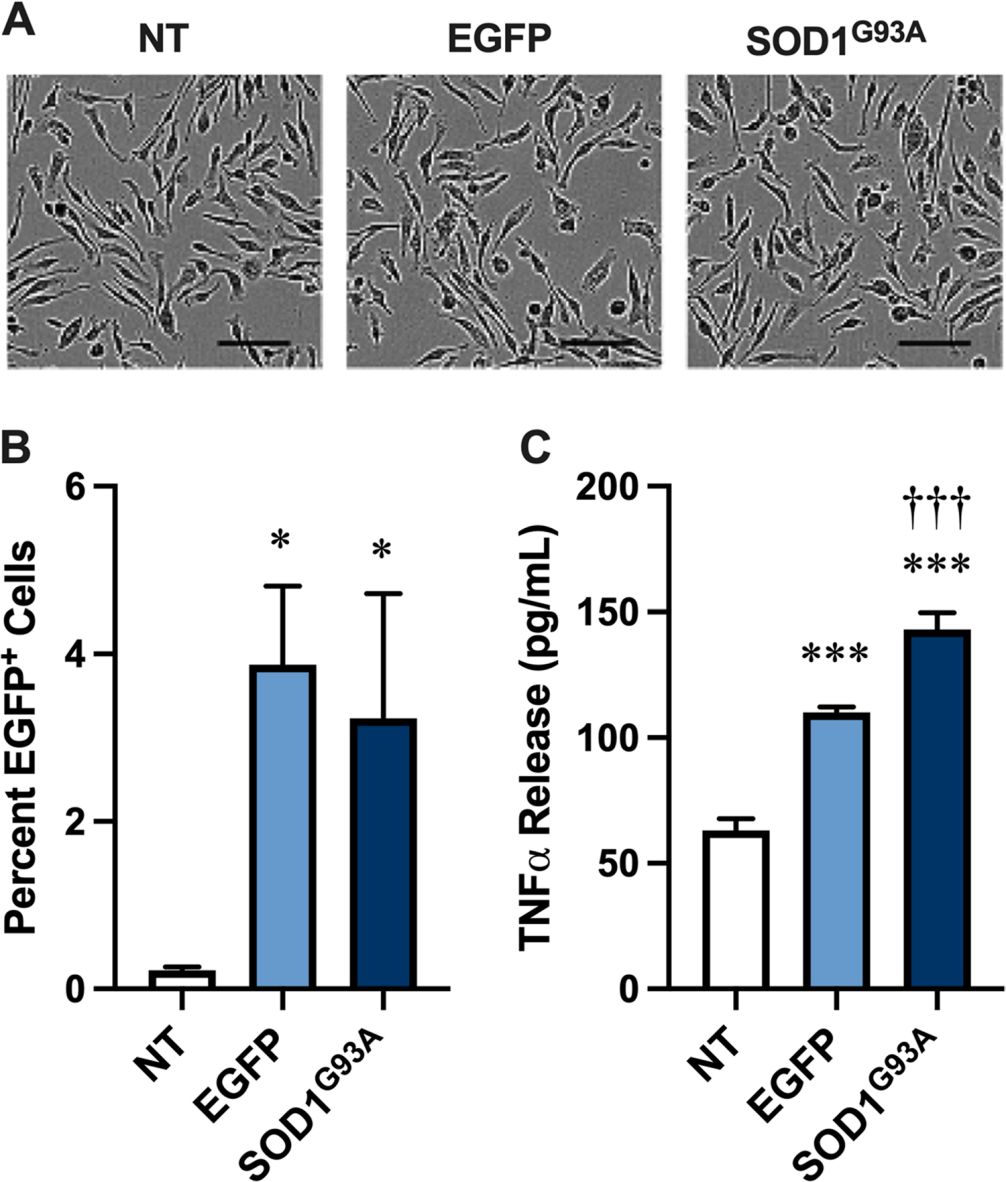
SOD1^G93A^ constitutively released from NSC-34 motor neurons associates with EOC13 microglia to promote TNFα release. **A**–**C** EOC13 cells were incubated with pelletable fractions from conditioned media of non-transfected (NT), or EGFP- or SOD1^G93A^-EGFP (SOD1^G93A^)-transfected NSC-34 cells. At 24 h, **A** cells were visualised by differential interference contrast microscopy, **B** the percentage of EGFP^+^ cells was assessed by flow cytometry, and **C** the concentration of TNFα released into cell supernatants was assessed by ELISA. **A** Data are representative of *n* = 6 experiments; bars represent 100 µm. **B**, **C** Data are presented as mean ± SD (*n* = 3); *p < 0.05, ***p < 0.01 compared to NT, ^†††^*p* < 0.01 compared to EGFP

Recombinant SOD1^G93A^ aggregates can be internalised by murine EOC13 microglia and induce TNFα release from these cells over 24 h [17]. Therefore, to determine if SOD1^G93A^-EGFP released from NSC-34 cells could act in a similar fashion to recombinant SOD1^G93A^ aggregates, EOC13 cells were incubated for 24 h in the presence of pelletable fractions obtained by centrifugation (20,000 × *g* for 30 min) of conditioned media (72 h) from non-transfected, and EGFP and SOD1^G93A^-EGFP transfected NSC-34 cells. Phase-contrast microscopy revealed similar morphology of EOC13 cells incubated with each pelletable fraction (Fig. 2A). Flow cytometric analysis revealed that the percent of EOC13 cells positive for EGFP fluorescence was negligible in those incubated with non-transfected pelletable fractions (Fig. 2B). In contrast, a small proportion of EOC13 cells incubated with either EGFP or SOD1^G93A^-EGFP pelletable fractions were positive for EGFP fluorescence (Fig. 2B) indicating that both EGFP and SOD1^G93A^-EGFP from NS34 cells associated with EOC13 microglia. Finally, ELISA measurements of media from EOC13 cells revealed that incubation with EGFP and SOD1^G93A^-EGFP pelletable fractions of conditioned media (72 h) induced significantly greater TNFα release compared to incubation with non-transfected pelletable fractions (Fig. 2C). Notably, TNFα release from EOC13 microglia incubated with SOD1^G93A^-EGFP pelletable fractions was statistically different than that of cells incubated with EGFP pelletable fractions. TNFα was absent in pelletable fractions from transfected NSC-34 cells (not shown). The relatively small amount of TNFα release from EOC13 microglia incubated with non-transfected pelletable fractions suggests that molecules present within the conditioned medium of non-transfected NSC-34 cells can also stimulate TNFα release from microglia, consistent with the presence of immune mediators in untreated NSC-34 cells [38]. Collectively these results indicate that SOD1^G93A^ released from NSC-34 cells can associate with and promote TNFα release from EOC13 cells.

### NSC-34 motor neurons express functional P2X7

**Fig. 3:**
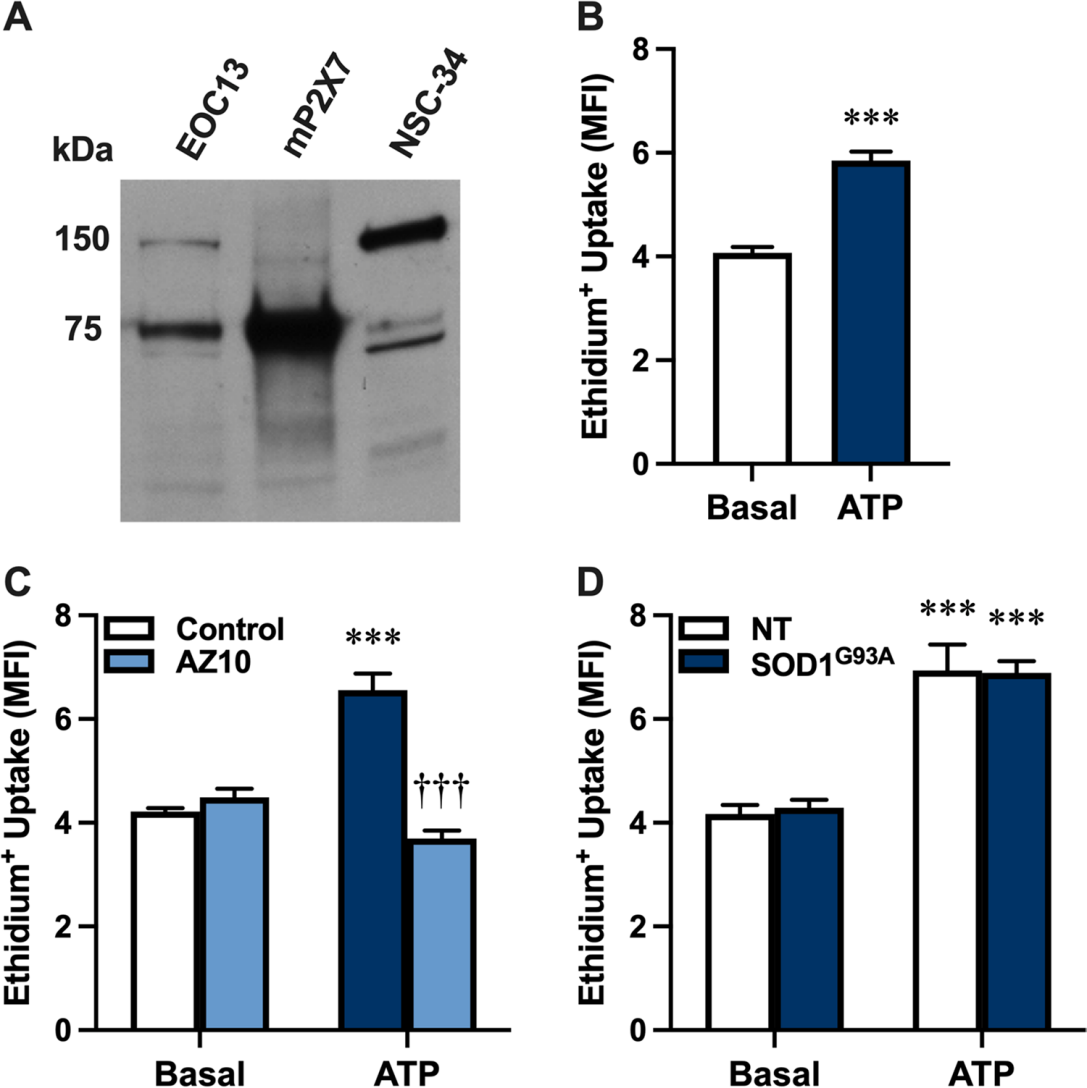
NSC-34 motor neurons express functional P2X7. **A** P2X7 expression in whole lysates of EOC13, murine P2X7-transfected HEK-293 (mP2X7) and NSC-34 cells was assessed by immunoblotting using an anti-P2X7 antibody. Data is representative of *n* = 3 experiments. **B**–**D** Non-transfected (NT) or **D** SOD1^G93A^-EGFP (SOD1^G93A^)-transfected NSC-34 cells were pre-incubated in the **B**–**D** absence or **C** presence of 10 μM AZ10606120 (AZ10) for 1 h. Cells were then incubated with 25 μM ethidium bromide in the absence (basal) or presence of 5 mM ATP for 20 min, and the reactions were stopped by the addition of 20 mM MgCl_2_. The MFI of ethidium^+^ uptake into cells was assessed by flow cytometry. Data are presented as mean ± SD (*n* = 3); ***p < 0.01 compared to corresponding basal, ^†††^*p* < 0.01 compared to ATP in the absence of AZ10

P2X7 has an emerging role in ALS [22, 23], and its activation by extracellular ATP can induce the release of various proteins from different cell types [28–30]. Other studies also indicate that primary rat motor neurons express P2X7 [39, 40]. Thus, it was hypothesised that NSC-34 motor neurons express P2X7, and that activation of this receptor could mediate SOD1^G93A^ release from these cells. To determine if P2X7 is present on NSC-34 motor neuron cells [32], whole lysates of NSC-34 cells, as well as murine EOC13 cells [34] and murine P2X7-transfected HEK-293 cells [41] (as positive controls) were examined by immunoblotting using an anti-P2X7 antibody. Immunoblotting revealed a minor band at 75 kDa in NSC-34 cell lysates, corresponding to the predicted size of glycosylated, monomeric P2X7, as well as major bands at 150 and 65 kDa corresponding to the predicted size of dimeric and non-glycosylated P2X7, respectively (Fig. 3A). Notably, the intensity of the 75 kDa band in NSC-34 cell lysates was lower than that of EOC13 and P2X7-transfected HEK-293 cells.

To determine whether NSC-34 cells expressed functional P2X7, ATP-induced ethidium^+^ uptake was assessed by flow cytometry. Incubation with 5 mM ATP induced significant ethidium^+^ uptake into NSC-34 cells compared to cells incubated in the absence of ATP (basal) (Fig. 3B). Moreover, pre-incubation of NSC-34 cells with 10 µM AZ10606120, a specific P2X7 antagonist [42], prior to incubation with 5 mM ATP completely blocked ATP-induced ethidium^+^ uptake (Fig. 3C). To determine whether transfection with SOD1 alters P2X7 activity in NSC-34 neurons, cells were transiently transfected with SOD1^G93A^-EGFP and ATP-induced ethidium^+^ uptake compared to non-transfected NSC-34 cells. ATP induced similar and significant ethidium^+^ uptake into both transfected and non-transfected cells (Fig. 3D). Collectively, these results indicate that NSC-34 cells express functional P2X7 and that the activity of this receptor in these cells is not altered by transient transfection with SOD1^G93A^-EGFP.

### P2X7 activation mediates SOD1^G93A^ release from NSC-34 motor neurons

**Fig. 4:**
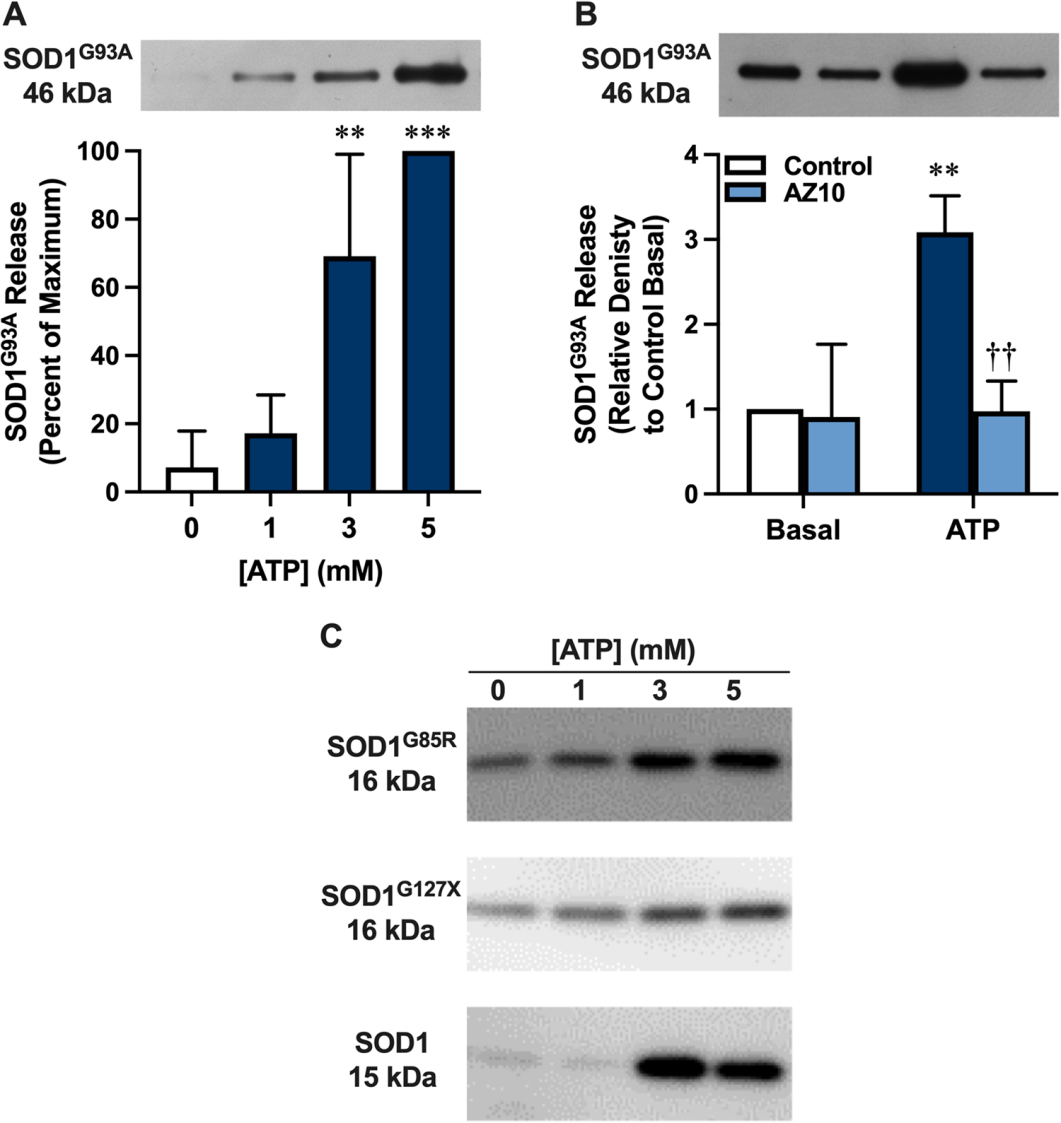
P2X7 activation mediates SOD1^G93A^ release from NSC-34 motor neurons. **A**, **B** SOD1^G93A^-EGFP (SOD1^G93A^)-transfected NSC-34 cells, or **C** non-EGFP SOD1^G85R^-, non-EGFP SOD1^G127X^- or non-transfected (SOD1) NSC-34 cells were pre-incubated in the **A**, **C** absence or **B** presence of 10 μM AZ10606120 (AZ10) for 1 h. Cells were then incubated in the absence (0 mM or basal as indicated) or presence of **A–C** 1, 3 or **A**–**C** 5 mM ATP for 20 min and pelletable fractions obtained by centrifugation. **A**, **B** The presence of SOD1 in pelletable fractions was assessed by immunoblotting using an anti-EGFP antibody and densitometry. One representative immunoblot (upper image) is shown, and data (lower image) are presented as mean ± SD (*n* = 3) relative to **A** maximum release or **B** release in the absence of AZ10 and ATP; **p < 0.02, ***p < 0.01 compared to 0 mM ATP or corresponding basal, ^††^*p* < 0.02 compared to ATP in the absence of AZ10. **C** The presence of SOD1 in pelletable fractions was assessed by immunoblotting using an anti-SOD1 antibody. Data are representative of *n* = 2–3 experiments

To determine if P2X7 activation could induce SOD1^G93A^ release, NSC-34 cells transiently transfected with SOD1^G93A^ (72 h) were incubated in the absence or presence of ATP (1, 3, or 5 mM) and the presence of SOD1^G93A^-EGFP in pelletable fractions obtained by centrifugation (20,000 × g for 30 min) examined by immunoblotting using an anti-EGFP antibody. Incubation with either 3 or 5 mM, but not 1 mM, ATP induced significant release of SOD1^G93A^-EGFP from cells compared to cells incubated in the absence of ATP (Fig. 4A). This ATP-induced release of SOD1^G93A^-EGFP corresponded to a parallel increase in total protein (Fig. S1), indicating that the ATP effect was not limited to SOD1^G93A^-EGFP. Pre-incubation of SOD1^G93A^-EGFP-transfected NSC-34 cells with 10 µM AZ10606120 prior to incubation with 5 mM ATP completely blocked ATP-induced SOD1^G93A^-EGFP release (Fig. 4B).

SOD1^G93A^ can be associated with fibrillar structures that can be macropinocytosed by motor neurons [11] or activate microglia [17]. Therefore, SOD1^G93A^-EGFP transfected NSC-34 cells were incubated in the absence or presence of 5 mM ATP and the pelletable fractions examined by transmission electron microscopy (TEM). Fibrillar structures, ranging from 100 to 700 nm, were identified in fractions from cells incubated in the presence of ATP but were largely absent in corresponding fractions from cells incubated in the absence of ATP (Fig. S2). To determine if the SOD1^G93A^-EGFP released following P2X7 activation co-localised with these fibrillar structures, pelletable fractions were examined by TEM and immunogold labelling with an anti-EGFP antibody. SOD1^G93A^-EGFP, identified by round black gold particles (indicated by arrows), were present alone and in association with fibrillary-like structures (Fig. S3). No immunogold labelling was evident in the corresponding isotype control (Fig. S3).

To determine if ATP could also induce the release of other forms of SOD1, NSC-34 cells, transiently transfected with plasmid DNA encoding non-EGFP-tagged SOD1^G85R^, non-EGFP-tagged SOD1^G127X^ or murine SOD1, were incubated in the absence or presence of ATP (1, 3, or 5 mM) and the presence of SOD1 in pelletable fractions examined by immunoblotting using an anti-SOD1 antibody. As for SOD1^G93A^-EGFP, both 3 mM and 5 mM ATP induced the release of SOD1^G85R^ and SOD1^G127X^ from transfected cells, as well as murine SOD1 from non-transfected cells (Fig. 4C). Together, this data indicates that P2X7 activation mediates the release of SOD1^G93A^ (and other forms of SOD1) from NSC-34 cells.

### P2X7-mediated SOD1^G93A^ release from NSC-34 motor neurons coincides with membrane blebbing

**Fig. 5:**
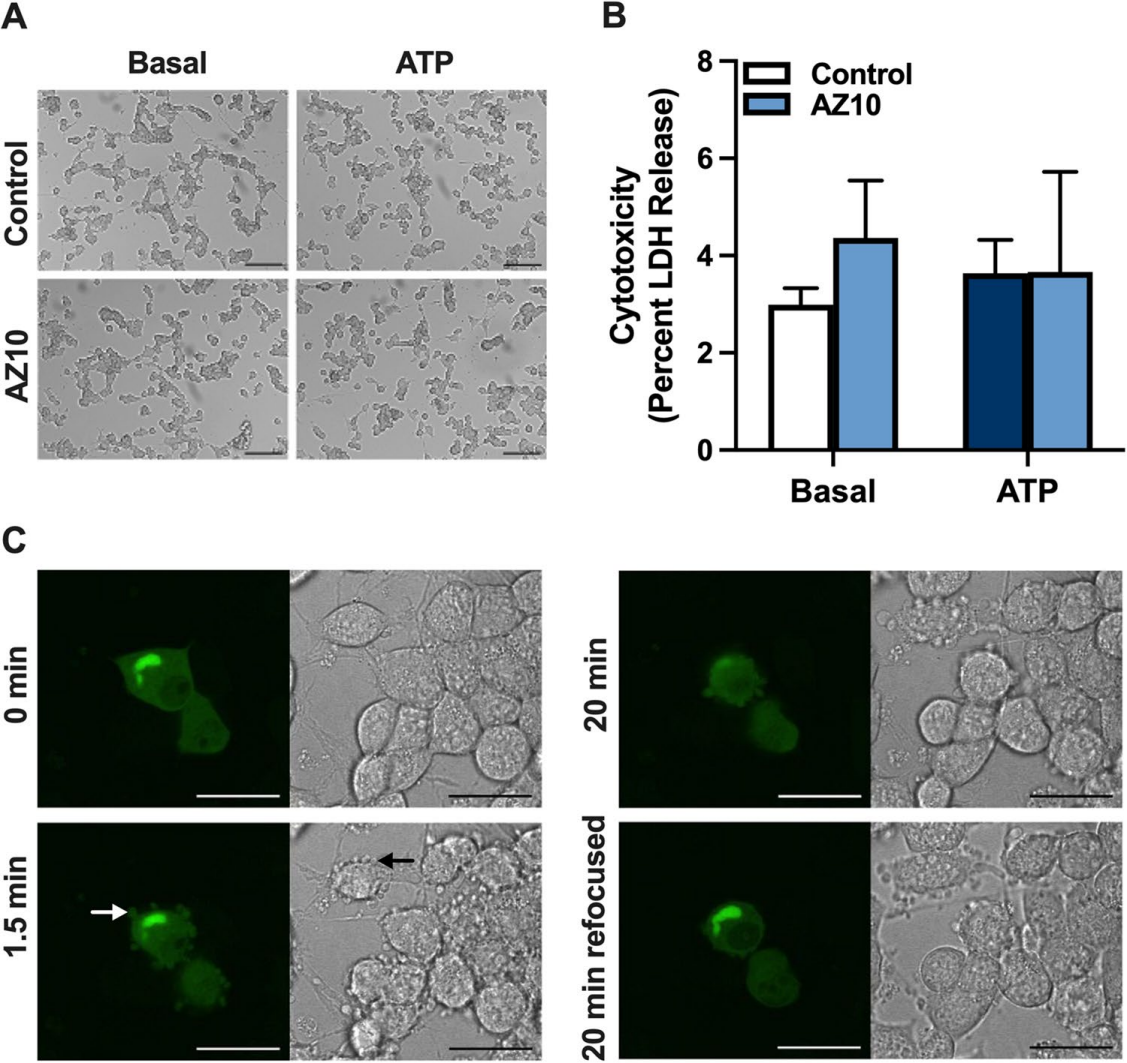
P2X7-mediated SOD1^G93A^ release from NSC-34 motor neurons coincides with membrane blebbing. **A**–**C** SOD1^G93A^-EGFP (SOD1^G93A^)-transfected NSC-34 cells were pre-incubated in the **A**–**C** absence or **A**, **B** presence of 10 μM AZ10606120 (AZ10) for 1 h. Cells were then incubated in the absence (basal) or presence of 5 mM ATP for 20 min. **A** Cell morphology was visualised by differential interference contrast microscopy. Data are representative of *n* > 20 fields of view; bars represent 100 µm. **B** Percent of cytotoxicity (LDH release) was determined using the Cytotoxicity Detection KitPLUS. Data are presented as mean ± SD (*n* = 3). **C** Membrane blebbing at 0, 1.5, and 20 min was visualised by fluorescent (left images) and bright-field (right images) microscopy. Refocusing was used to examine intracellular SOD1^G93A^-EGFP at 20 min (20 min refocused). Data are representative of *n* = 6 experiments; white and black arrows indicate an example of a membrane bleb in transfected and non-transfected cells, respectively; bars represent 25 µm

P2X7 activation can induce cytotoxicity under certain circumstances [43] raising the possibility that P2X7-mediated SOD1^G93A^ release from NSC-34 cells occurs via cell death. Therefore, SOD1^G93A^-EGFP transfected NSC-34 cells were incubated as above and assessed for cell death by microscopy and LDH release. Incubation of cells in the absence (basal) or presence of ATP revealed no morphological differences, with most cells displaying discrete cell bodies with short, spindled shaped processes (Fig. 5A). Moreover, pre-incubation of cells in the absence or presence of AZ10606120 revealed no morphological differences (Fig. 5A). Similarly, cells incubated under the same conditions resulted in no significant differences in LDH release between treatments, with less than 6% of the total cellular LDH released in each treatment group (Fig. 5B).

P2X7 activation can induce membrane blebbing in various cell types [44–46], and membrane blebbing can be associated with protein release [47]. Therefore, to determine a role for membrane blebbing in P2X7-mediated SOD1 release, SOD1^G93A^-EGFP transfected NSC-34 cells were incubated as above and membrane blebbing examined via confocal microscopy with fluorescent and bright field images collected every 30 s for 20 min (Fig. S4). Morphological changes consistent with membrane blebbing were observed within 1.5 min of ATP addition (Fig. 5C). Membrane blebs ranged in size from 1 to 3 µm and occurred in both SOD1^G93A^-EGFP expressing (white arrow) and non-expressing (black arrow) cells (Fig. 5C). Moreover, SOD1^G93A^-EGFP appeared to be released in blebs, evident from the presence of EGFP signal, while the larger mass of cytosolic SOD1^G93A^-EGFP (identified as EGFP^+^ foci) appeared to remain inside cells following intracellular refocusing at 20 min (Fig. 5C). Collectively, these results demonstrate that P2X7-mediated SOD1^G93A^ release from NSC-34 motor neurons coincides with membrane blebbing.

### SOD1^G93A^ released from NSC-34 motor neurons via P2X7 activation associates with NSC-34 motor neurons and EOC13 microglia to promote ER stress and TNFα release, respectively

**Fig. 6:**
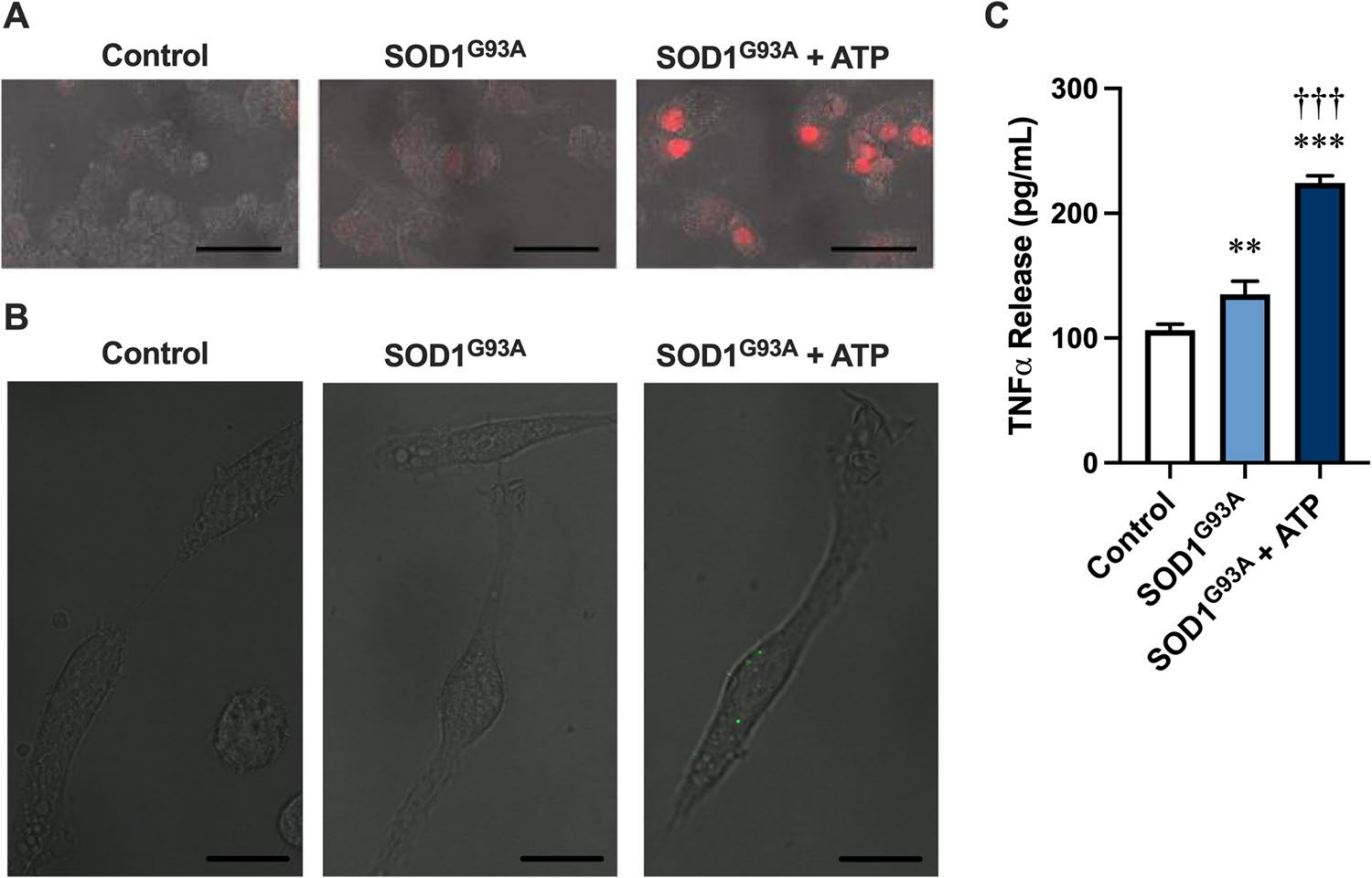
SOD1^G93A^ released from NSC-34 motor neurons via P2X7 activation associates with NSC-34 motor neurons and EOC13 microglia to promote ER stress and TNFα release, respectively. **A**–**C** SOD1^G93A^-EGFP-transfected NSC-34 cells were incubated in the absence (SOD1^G93A^) or presence of 5 mM ATP (SOD1^G93A^ + ATP) for 20 min, and pelletable fractions obtained by centrifugation and used as follows. **A** XBP1-tdTomato-transfected NSC-34 cell were incubated with medium alone (Control) or pelletable fractions (SOD1^G93A^ or SOD1^G93A^ + ATP). At 24 h, cellular XBP1-tdTomato fluorescence was visualised by confocal microscopy. Data are representative of *n* = 2 experiments; bars represent 50 µm. **B**, **C** EOC13 cells were incubated with medium alone (control) or pelletable fractions (SOD1^G93A^ or SOD1^G93A^ + ATP). **B** At 2 h, EGFP fluorescence was visualised by confocal microscopy. Data are representative of *n* = 6 experiments; bars represent 15 µm. **C** At 24 h, the concentration of TNFα released into cell supernatants was assessed by ELISA. Data are presented as mean ± SD (*n* = 3); **p < 0.02, ***p < 0.01 compared to control, ^†††^*p* < 0.01 compared to SOD1^G93A^

To determine if SOD1^G93A^-EGFP released from transfected NSC-34 motor neurons via P2X7 activation could associate with naïve NSC-34 cells and induce ER stress, SOD1^G93A^-EGFP transfected NSC-34 cells were incubated in the absence or presence of 5 mM ATP, and pelletable fractions obtained by centrifugation (20,000 × *g* for 30 min). Naïve XBP1-tdTomato-transfected NSC-34 cells were then incubated for 24 h in the presence of complete medium (negative control) or pelletable fractions suspended in complete medium. Confocal microscopy revealed a greater proportion of XBP1-tdTomato fluorescence in NSC-34 cells incubated with ATP-induced SOD1^G93A^-EGFP pelletable fractions compared to cells incubated with complete culture medium or SOD1^G93A^-EGFP pelletable fractions, both of which had negligible or low amounts of fluorescence, respectively (Fig. 6A).

Finally, to determine if SOD1^G93A^-EGFP released from transfected NSC-34 cells via P2X7 activation could associate with EOC13 microglia and induce TNFα release, EOC13 cells were incubated for up to 24 h in the presence of complete medium or pelletable fractions as above. Confocal microscopy revealed the presence of EGFP associated with EOC13 cells incubated with ATP-induced SOD1^G93A^-EGFP pelletable fractions but not in cells incubated with complete medium or SOD1^G93A^-EGFP pelletable fractions at 2 h (Fig. 6B). ELISA measurements at 24 h revealed that EOC13 cells incubated with ATP-induced SOD1^G93A^-EGFP pelletable fractions EGFP induced significantly greater TNFα release compared to cells incubated with complete medium or SOD1^G93A^-EGFP pelletable fractions (Fig. 6C). Together, the above data demonstrate that SOD1^G93A^ released from NSC-34 motor neurons via P2X7 activation associates with both NSC-34 motor neurons and EOC13 microglia to promote ER stress and TNFα release, respectively.

## Discussion

Accumulating evidence suggests a role for mutant SOD1 in the prion-like propagation in ALS [4]; however, the biological impacts of this process and the precise mechanisms of SOD1 release remain to be fully resolved. This study demonstrated that aggregated SOD1^G93A^ released from NSC-34 motor neurons could be transmitted to naïve NSC-34 cells and murine EOC13 microglia to induce ER stress and TNFα release, respectively. Moreover, this study demonstrated that NSC-34 cells express P2X7 and that activation of this receptor can induce the release of aggregated SOD1^G93A^ from these cells. Furthermore, this released material could associate with and induce ER stress in NSC-34 cells and TNFα release from EOC13 cells. A limitation regarding the role of P2X7 in NSC-34 cells is that the current study relied on the use of a single P2X7 antagonist, AZ1060120. Although this antagonist is a well-known high affinity, potent and selective P2X7 antagonist [42], which binds the allosteric binding pocket of this receptor [48, 49], additional studies using a second P2X7 antagonist or *P2rx7* gene silencing are required to confirm the current observations. Moreover, the current study relied on the use of a single P2X7 agonist, ATP. The P2X7 agonist, 2'(3')-O-(4-benzoylbenzoyl) ATP was not tested due to its potential to activate other P2X receptor subtypes [50].

The major finding of the current study is that P2X7 activation by extracellular ATP induces the release of SOD1^G93A^ aggregates from NSC-34 cells transiently transfected with SOD1^G93A^, which can be subsequently transmitted to naïve NSC-34 and EOC13 cells to potentially propagate disease. These findings were extended to SOD1^G85R^ and SOD1^G127X^ from transfected cells, as well as murine SOD1 from non-transfected cells, with apparent differences in the amount of SOD1 aggregates released most likely reflecting differences in SOD1 expression or aggregation. Collectively, to the best of our knowledge, this is the first description of a receptor-mediated pathway of aggregated SOD1 release. It is currently thought that SOD1 aggregates are released directly from dead or dying cells or via exosomes from live cells and cells early in the dying process [51]. Thus, P2X7-mediated release provides an alternate but not mutually exclusive mechanism of aggregated SOD1 release. Measurements of LDH release suggest that the P2X7-mediated release of aggregated SOD1 occurred independently of cell death. Similarly, others have shown that P2X7 agonists do not induce death of SOD1^G93A^- or SOD1^WT^-transfected NSC-34 cells [52]. However, given that P2X7 activation can induce the death of rat motor neurons [39, 40], it cannot be excluded that P2X7 mediated the release of aggregated SOD1 from cells early in the dying process. In this regard, P2X7 activation on NSC-34 cells transiently transfected with SOD1^G93A^ was associated with membrane blebbing, which can be associated with cell death in some [53] but not all instances [54, 55]. It is also possible that P2X7 activation may mediate SOD1 release to remove excess amounts of this protein, given that P2X7 activation can promote autophagy in SOD1^G93A^ mice [56, 57]. In contrast, it is unlikely that the increase in aggregated SOD1 release following P2X7 activation was due to increased SOD1 synthesis. To the best of our knowledge, the impact of P2X7 activation on SOD1 synthesis is unknown, but the effect of P2X7 activation was studied over a relatively short period (20 min), while the turnover of SOD1 in vitro and in vivo is slow (hours to days) [58, 59]. Other possible mechanisms of aggregated SOD1 release following P2X7 activation include direct exocytosis or exocytosis of extracellular vesicles (ectosomes, exosomes, and/or lysosomes/autophagosomes), mechanisms common to both P2X7 activation [60] and SOD1 release in other contexts [61].

The current study demonstrates that aggregated SOD1^G93A^ released either constitutively or following P2X7 activation can subsequently be transmitted to naïve NSC-34 and EOC13 cells to induce ER stress and TNFα release, respectively. ER stress, resulting from the accumulation of misfolded or unfolded proteins, can stimulate the unfolded protein response, which can eventually result in motor neuron dysfunction and death [62]. Notably, ER stress is found in the motor neurons that degenerate first in SOD1^G93A^ mice, which subsequently influences disease progression [63]. This earlier finding highlighted the contribution of ER stress to the initiation of ALS. However, accumulating evidence also indicates important roles for ER stress in the progression of ALS at later time points [62]. Likewise, TNFα can also contribute to ALS progression, although the contributions of this cytokine to the pathophysiology of ALS remain varied and controversial [64]. Nevertheless, increased release of TNFα may contribute to neuroinflammation, glutamate-mediated excitotoxicity and muscle wasting in this disease [64]. Combined, these observations are consistent with the notion that aggregated SOD1^G93A^ release and transmission to other motor neurons and microglia may facilitate the progression of ALS.

This study demonstrates for the first-time the presence of functional P2X7 on NSC-34 cells. Although the presence of P2X7 on neurons is controversial [65, 66], P2X7 has been observed in mouse and rat motor neurons in situ [67, 68], and activation of these receptors on these cells can induce vesicle exocytosis [67] and, as mentioned above, cell death [39, 40]. However, it should be noted that NSC-34 cells were obtained by fusing N18TG-2 neuroblastoma cells with murine spinal cord cells [32] and that N18TG-2 cells have been shown to express functional P2X7 [69]. Thus, the possibility remains that the presence of P2X7 on NSC-34 cells is acquired from the neuroblastoma cell line rather than spinal cord cells. This caveat notwithstanding, the current study supports the notion that P2X7 activation may mediate the release and transmission of aggregated SOD1 in vivo. Moreover, it is hypothesised that this mechanism may be a target of the central nervous system (CNS)-penetrant P2X7 antagonists JNJ-47965567 [22, 70] or AXX71 [56], which can slow disease progression in ALS SOD1^G93^A mice in a dose-dependent manner. Whether the poorly CNS-penetrant P2X7 antagonist Brilliant Blue G, which likewise slows disease progression in ALS SOD1^G93A^ mice [71], also impairs P2X7-mediated aggregated SOD1 release remains unknown.

The murine *P2rx7* gene is polymorphic, with many mouse strains encoding the well-established loss-of-function polymorphism, P451L [72, 73]. Despite this, the *P2rx7* genotype of NSC-34 or EOC13 cells is unknown. EOC13 cells were derived from a C3H mouse [74], a strain that encodes the 451L allele [75]. Thus, EOC13 cells are likely to encode the 451L allele and express P2X7 with reduced activity. However, the *P2rx7* genotype of NSC-34 cells is more difficult to ascertain due to their complex origin. As noted above, NSC-34 cells were obtained by fusing N18TG-2 neuroblastoma cells with murine spinal cord cells [32]. This is further complicated with N18TG-2 cells being derived from C1300 tumour cells [76], reportedly from A/J mice [77], a strain that encodes the P451 allele [75]. While the spinal cord cells, used to generate NSC-34 cells, were obtained from CD-1 mice [32], a strain of unknown *P2rx7* genotype. CD-1 mice however display P2X7 properties consistent with mice encoding the P451 allele [75]. Thus, NSC-34 cells are likely to encode the P451 allele and express P2X7 with normal activity. Despite the 451L allele encoding for a loss-of-function, the ATP sensitivity of P2X7 containing either a proline or leucine residue at position 451 are similar [78, 79]. Notably, the ATP sensitivity of P2X7 in N18TG-2 cells (EC_50_ of 1.8 mM) is at least twofold greater [69] than that of recombinant murine P2X7 overexpressed in HEK-293 cells (EC_50_ of 0.7 mM) [80]. Thus, the aforementioned requirement for relatively high ATP concentrations (≥ 3 mM) to active P2X7 in NCS-34 cells may reflect low P2X7 expression (paralleling the P2X7 immunoblotting data above) or the presence of another, unknown polymorphism in the cells.

In addition to the use of a single P2X7 agonist (ATP) and antagonist (AZ1060120), this study includes other limitations. First, relatively high ATP concentrations (≥ 3 mM) were required to promote aggregated SOD1^G93A^ release from NSC-34 cells. It is unknown if ATP is released and at what concentrations in ALS. However extracellular ATP concentrations are thought to dramatically increase in the CNS under pathological conditions but measuring this directly in vivo remains challenging [81]. Second, there is a possibility that ATP was present in the pelletable fractions obtained following incubation with ATP and this may have mediated the observed effects in naïve NSC-34 or EOC13 cells via activation of P2X7. However, this appears improbable as the pelletable fractions were acquired by centrifugation at 20,000 × *g* for 30 min, are unlikely to contain residual ATP, a soluble molecule with a molecular weight of 507 Da, with most if not all of the ATP presumably residing in the supernatant. In support of this notion, SOD1 constitutively released from NSC-34 cells caused similar effects in naïve NSC-34 or EOC13 cells to that caused by ATP-induced SOD1. There is the possibility that very low concentrations of ATP, which could activate other P2 receptors potentially present in NSC-34 or EOC13 cells, exist in the pelletable fractions, but the presence of other P2 receptors in these cell types is unknown. Third, the possibility remains that transfection of NSC-34 cells may have induced pro-inflammatory effects to aid the observed effects in naïve NSC-34 or EOC13 cells. For example, others have reported that transfection of NSC-34 cells with mutant SOD1 can cause the release of exosomes containing inflammatory-related microRNAs [82] or the upregulation of cytosolic phospholipase A2α in TNFα-dependent manner [83]. The induction of such pro-inflammatory effects may offer an explanation as to why pelletable fractions from EGFP-transfected NSC-34 caused a small amount of TNFα release from naïve EOC13 cells.

**Fig. 7:**
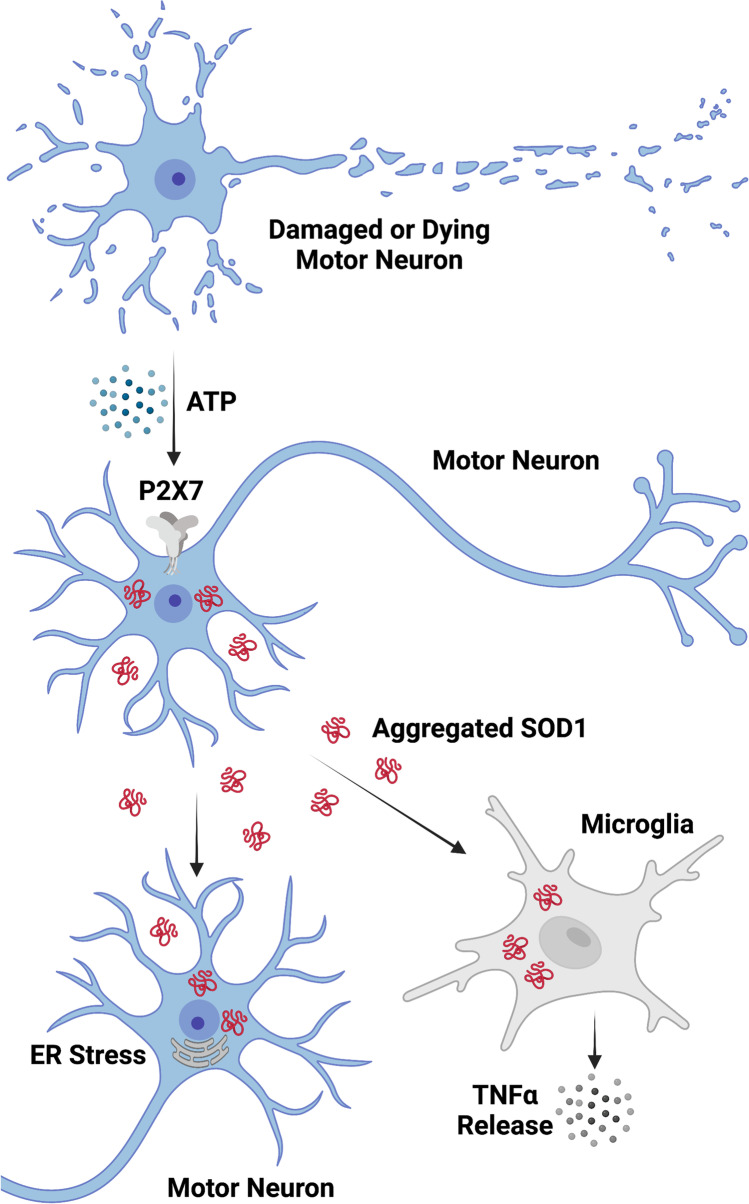
Role of the ATP-P2X7 signalling axis in the transmission of SOD1 aggregates and progression of ALS. Damaged or dying motor neurons (or activated glia; not shown) in ALS patients release ATP to stimulate P2X7 on neighbouring motor neurons leading to aggregated SOD1 release. Released SOD1 aggregates are internalised by other neighbouring motor neurons or microglia to promote ER stress or TNFα release, respectively, to potentially promote disease progression in ALS patients. Created with BioRender.com

In conclusion, the current study demonstrates that aggregated SOD1^G93A^ can be released constitutively or via P2X7 activation from NSC-34 cells, which can subsequently be transmitted to naïve NSC-34 and EOC13 cells to induce ER stress and TNFα release, respectively (Fig. 7). Collectively, this provides further support for the prion-like propagation of SOD1 in ALS and a possible explanation for the therapeutic benefits observed with P2X7 antagonism in ALS SOD1^G93A^ mice.

## Supplementary Information

Below is the link to the electronic supplementary material.

Supplementary file1 (AVI 30723 KB)

Supplementary file2 (AVI 30723 KB)

Supplementary file3 (AVI 30723 KB)

Supplementary file4 (PDF 2049 KB)

## Data Availability

Data is contained within the article or supplementary material. Data not shown is available from the corresponding authors, upon reasonable request.
