# Supplementary material for: P2X7 receptor activation mediates superoxide dismutase 1 (SOD1) release from murine NSC-34 motor neurons
Source: Purinergic Signal. 2022 Apr 28;18(4):451–67. doi: 10.1007/s11302-022-09863-5 (PMC9832181; doi:10.1007/s11302-022-09863-5)
Supplement: Supplementary file 4 — Supplementary file4 (PDF 2049 KB) [file 11302_2022_9863_MOESM4_ESM.pdf]

Supplementary Material

P2X7 receptor activation mediates superoxide dismutase 1 (SOD1) release from murine NSC-34 motor neurons

Rachael Bartlett [1,2], Diane Ly [1,2], Neil R. Cashman [3], Ronald Sluyter [1,2], Justin J. Yerbury [1,2]

[1] Illawarra Health and Medical Research Institute, Wollongong, NSW, 2522, Australia

[2] Molecular Horizons and School of Chemistry and Molecular Bioscience, University of Wollongong, Wollongong, NSW, 2522, Australia

[3] Djavad Mowafaghian Centre for Brain Health, University of British Columbia, Vancouver, BC, V6T 1Z3, Canada

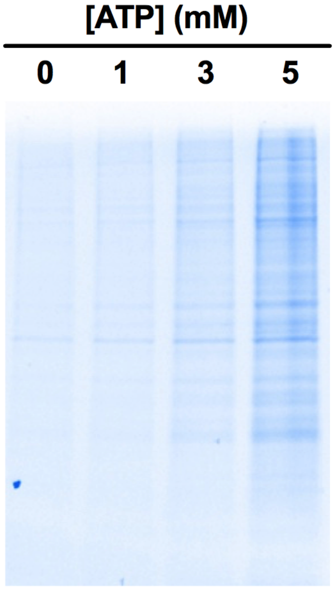

Fig. S1 Extracellular ATP mediates protein release from NSC-34 motor neurons. SOD1^{G93A}-EGFP (SOD1^{G93A})-transfected NSC-34 cells were pre-incubated for 1 h and then in the absence (0 mM) or presence of 1, 3 or 5 mM ATP for 20 min. Pelletable fractions were obtained by centrifugation, resuspended in equal volumes of sample buffer containing 5% 2-mercaptoethanol and separated using Stain-Free gels. The presence of total protein in each lane was captured using a Criterion Stain Free Gel Imaging System. One representative gel is shown. Data are representative of n = 3 experiments.

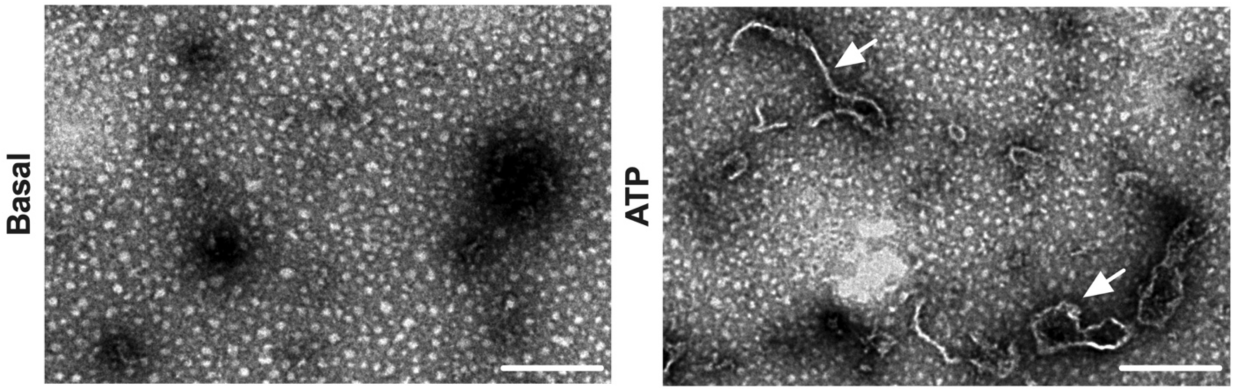

Fig. S2. P2X7 activation mediates SOD1^{G93A} release from NSC-34 motor neurons. SOD1^{G93A}-EGFP (SOD1^{G93A})-transfected NSC-34 cells were incubated in the absence (basal) or presence of 5 mM ATP for 20 min and pelletable fractions obtained by centrifugation. The presence of fibrillar SOD1 (arrows) in pelletable fractions was assessed by transmission electron microscopy. Data are representative of n = 1 experiment; bars represent 200 nm.

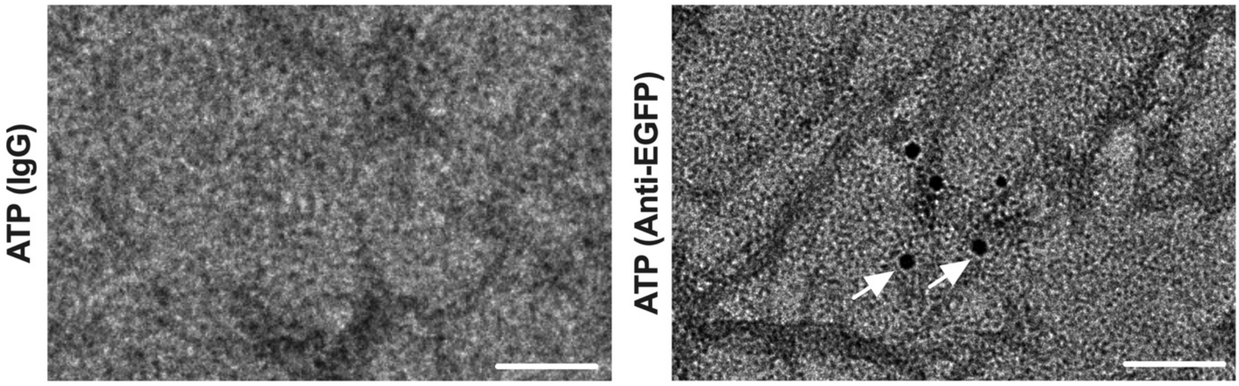

Fig. S3. P2X7 activation mediates SOD1^{G93A} release from NSC-34 motor neurons. SOD1^{G93A}-EGFP (SOD1^{G93A})-transfected NSC-34 cells were incubated in the presence of 5 mM ATP for 20 min and pelletable fractions obtained by centrifugation. Samples were pre-labelled with rabbit IgG or an anti-EGFP antibody and gold-conjugated secondary antibody and the presence of gold-labelled SOD1 (arrows) in pelletable fractions was assessed by transmission electron microscopy. Data are representative of n = 2 experiments; bars represent 40 nm.

Fig. S4 P2X7-mediated SOD1^{G93A} release from NSC-34 motor neurons coincides with membrane blebbing. SOD1^{G93A}-EGFP-transfected NSC-34 cells were incubated with 5 mM ATP and cells visualised by microscopy, with images captured every 30 s over 20 min. Representative recordings of (A) fluorescent, (B) bright-field and (C) overlayed images of cells within the same field of view are provided on the article site.
